# Cross‐sectional study of gender and ethnicity patterns in leadership in the American Academy of Dermatology

**DOI:** 10.1016/j.ijwd.2021.10.009

**Published:** 2021-11-02

**Authors:** Cindy E. Parra, Albert G. Wu, Shari R. Lipner

**Affiliations:** a Department of Dermatology, Weill Cornell Medicine, New York, New York; b New York Medical College School of Medicine, Valhalla, New York

**Keywords:** Ethnicity, gender, American Academy of Dermatology, AAD, leadership

*Dear Editors:*

 

**What is known about this subject in regard to women and their families?**

• Female and underrepresented minority physicians have historically been underrepresented in dermatology and academia, both in absolute terms and proportionally to their population percentage.
• In recent years, the number of female and underrepresented minority residents entering the field of dermatology have increased significantly.
• Multiple initiatives have been established by dermatology professional organizations to combat this underrepresentation, focusing on increasing awareness, funding, and mentorship of underrepresented populations.

**What is new from this article as messages for women and their families?**

• Over the last 10 years, underrepresented minority and female representation have significantly increased in the American Academy of Dermatology general membership.
• This change has been partially paralleled by an increase in underrepresented minority and female representation in American Academy of Dermatology leadership.
• However, this growth is smaller and has been mainly reflected in nonexecutive positions.

Dermatology is one of the least racially and ethnically diverse fields in the United States (Chen and Shinkai, 2017; Pandya et al., 2016). In 2018, ethnic minorities composed 33.3% of the U.S. population, but only 9.8% of board-certified dermatologists (Association of American Medical Colleges, 2019). Underrepresented minorities (URMs) composed only 7.4% of dermatology faculty in 2020 (Xierali et al., 2020), a statistically insignificant increase from 4.8% in 1970*.* With a new American Academy of Dermatology (AAD) 3-year initiative to improve diversity, equity, and inclusion, we aimed to analyze URM representation trends in AAD leadership positions and general membership.

We obtained leadership (president, vice president, board of directors, councils, committees, task forces) and membership ethnicity data from the AAD from 2010 to 2021. Extracted data were tabulated and analyzed with Excel Analysis Toolpak 6.0. URM encompassed African Americans, Hispanics, and Native Americans. Linear regression was used to model data directionality (α = .05).

Cumulatively, over the study period, available leadership positions increased from 284 to 371, and memberships increased from 17,322 to 20,746. Fifty-one percent of leadership and 29.9% of members reported their ethnicity. Total percentage of URMs elected/appointed to leadership positions increased from 4.2% (n = 12) to 19.1% (n = 71) from 2010 to 2021 (Pearson coefficient *r* = .68; Fig. 1), which was almost entirely from nonpresidential positions. URM board-certified members increased from 2.2% (n = 397) to 3.0% (n = 620; *r* = .95) between 2010 and 2020.

**Fig. 1:**
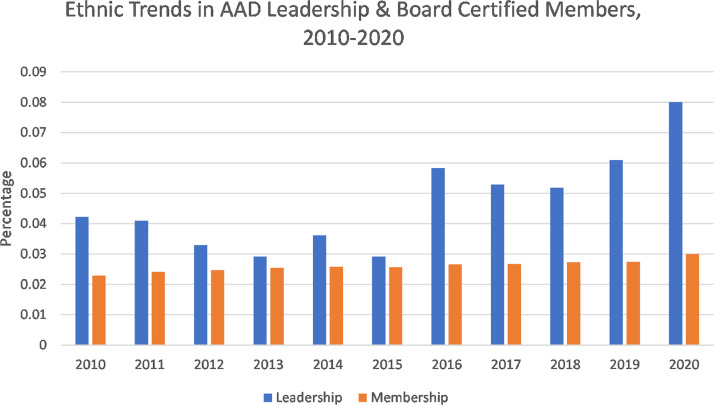
Trends in underrepresented minority representation of American Academy of Dermatology leadership and general members over time.

Our study demonstrated a positive trend in URM representation among AAD leadership, exceeding a smaller increase in URM AAD membership. Membership growth was likely due to increased numbers of URM board-certified dermatologists. Dermatology organizations have also identified and encouraged opportunities to increase the proportion of URM board-certified dermatologists (Pritchett et al., 2018). AAD's diversity efforts include promoting the Diversity Champions and Diversity Mentorship Programs and implementing a diversity roadmap to foster diversity in both its governance structures and the dermatology specialty overall. These trends are promising, but continued efforts are needed to encourage URM dermatologists to both join professional organizations and apply for leadership positions. The Women's Dermatologic Society has implemented a Diversity Task Force and has had a track record of having URM presidents. Increased reporting of dermatologists’ race/ethnicity demographics and understanding barriers to URMs joining and becoming leaders are critical to promoting diversity in professional organizations. Optimizing premedical and medical URM student recruitment, networking, and mentorship may foster interest in dermatology and encourage increased AAD URM representation.

Our study is subject to several limitations. Ethnicity self-reporting was incomplete in a notable proportion of membership data. However, there have been positive trends in AAD demographic reporting. The small number of leadership positions magnifies statistical differences.

Our data show growth of URM representation in both AAD leadership and membership between 2010 and 2020. We hope that our findings support the importance of continued efforts to achieve a physician workforce that reflects the patient populations that we serve. Given the unprecedented events of the past year and the need for accurate diagnoses in patients with skin of color, now is the time to openly discuss and address disparities in dermatology to thoughtfully improve the quality of care we deliver to all patients.

## Acknowledgments

The authors thank the American Board of Dermatology for providing data for this manuscript.

## Conflicts of interest

None.

## Funding

None.

## Study approval

The author(s) confirm that any aspect of the work covered in this manuscript that has involved human patients has been conducted with the ethical approval of all relevant bodies.
